# Steroid‐Induced Takotsubo Cardiomyopathy in a Patient With Immune Thrombocytopenic Purpura

**DOI:** 10.1002/ccr3.71047

**Published:** 2025-10-03

**Authors:** Qutaiba Qafisheh, Roaa Aljunaidi, Abdalhakim Shubietah, Mohammed AbuBaha, Hossam Salameh, Muath Baniowda, Mohanad Qwaider, Mohammad Alqadi, Imad Hariri

**Affiliations:** ^1^ Department of Medicine University of Toledo Toledo Ohio USA; ^2^ Faculty of Medicine and Health Sciences Palestine Polytechnic University Hebron Palestine; ^3^ Department of Medicine Advocate Illinois Masonic Medical Center Chicago Illinois USA; ^4^ Department of Medicine An‐Najah National University Nablus Palestine; ^5^ Department of Medicine University of Missouri–Kansas City Kansas City Missouri USA; ^6^ ProMedica Physician Group – Cardiology Toledo Ohio USA

**Keywords:** immune thrombocytopenic purpura (ITP), steroid cardiomyopathy, steroid induced Takotsubo, Takotsubo cardiomyopathy

## Abstract

This case report highlights a temporal relationship between dexamethasone administration and the onset of Takotsubo cardiomyopathy, underscoring the importance of recognizing pharmacologic agents as potential non‐traditional triggers, particularly in patients with underlying cardiovascular disease and no identifiable emotional or physical stressors. Diagnosis favored a dexamethasone‐triggered Takotsubo phenotype based on non‐obstructive coronaries, regional wall‐motion abnormalities extending beyond a single arterial territory, absence of alternate stressors, and biologic plausibility of steroid‐mediated catecholaminergic upregulation.

## Graphical abstract

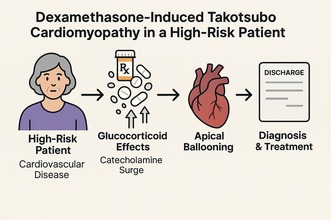

## 1 Introduction

Beyond classic emotional or physical stressors, iatrogenic pharmacologic triggers are increasingly recognized in Takotsubo cardiomyopathy (TCM). Exogenous catecholamines and β‐agonists are established precipitants, underscoring the central role of catecholaminergic excess in pathophysiology. Glucocorticoids can potentiate this axis: dexamethasone, via glucocorticoid‐receptor signaling in the adrenal medulla, upregulates catecholamine biosynthesis and increases epinephrine and norepinephrine in a dose‐dependent manner, providing biologic plausibility for steroid‐associated TCM in susceptible patients. Because prognosis and recurrence risk may vary by trigger phenotype, systematic elicitation of recent medication exposures is essential and may inform risk stratification and counseling. It is also important to note that the condition predominantly affects postmenopausal women, who represent the majority of reported cases [1, 2].

## 2 Case History/Examination

A 72‐year‐old female with a past medical history of immune thrombocytopenic purpura (ITP), coronary artery disease with a drug‐eluting stent placed in the left anterior descending (LAD) artery in 2009, non‐alcoholic fatty liver disease, Sjögren's syndrome, and hypertension presented to the emergency department with substernal chest pain persisting for 12–15 h. The pain radiated to her back, jaw, and both upper extremities.

## 3 Differential Diagnosis, Investigations and Treatment

**FIGURE 1:**
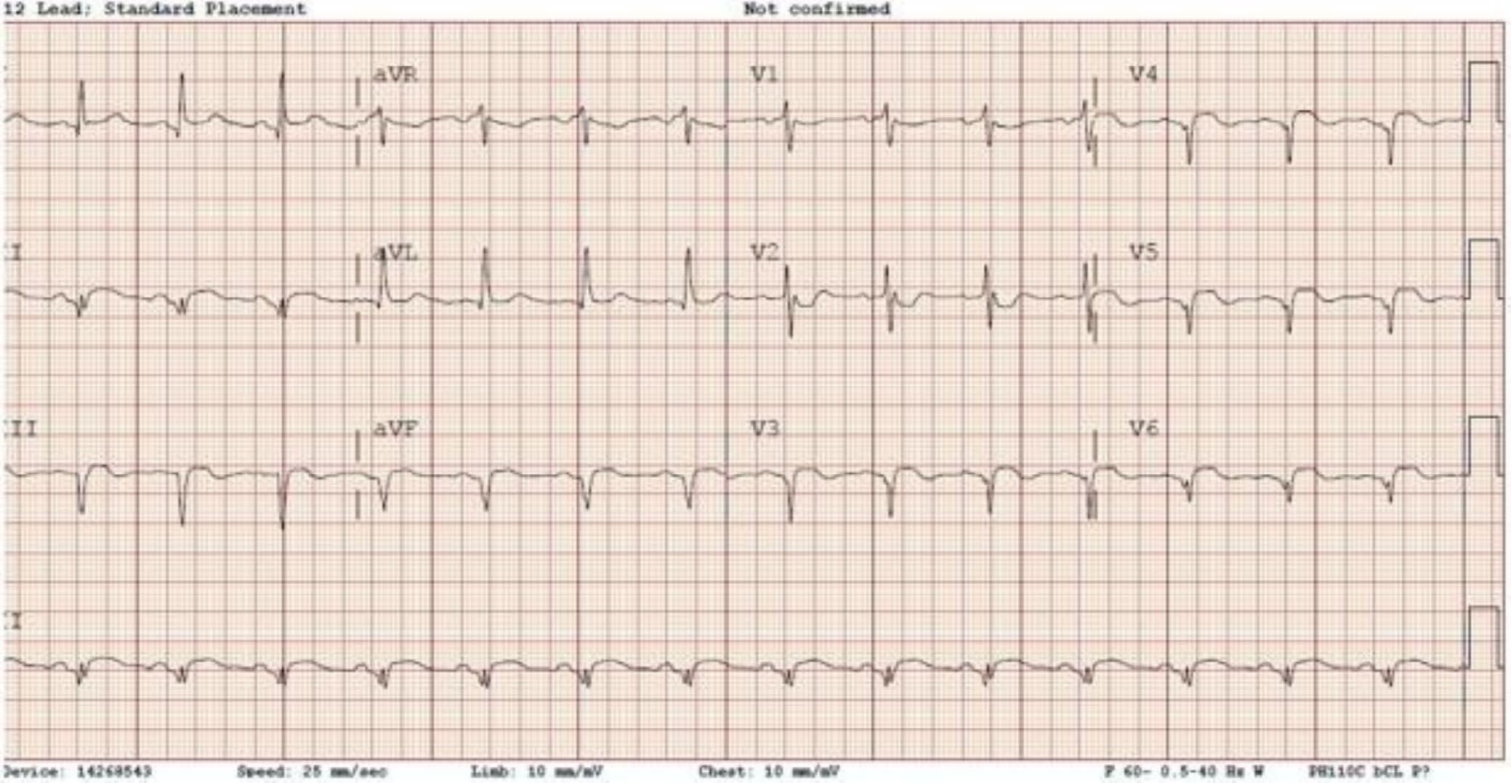
ST‐segment elevation and Q‐wave changes localized to the inferolateral leads on ECG.

The initial electrocardiogram (ECG) demonstrated ST‐segment elevation and Q‐waves in the inferolateral leads—approximately 2 mm in lead III, 1–1.5 mm in lead II, 1 mm in aVF, and 2 mm in leads V5–V6 (Figure 1)—findings that were not present on the baseline ECG. Her admission serum troponin I level was markedly elevated at 16.55 ng/mL (reference range: 0–0.04 ng/mL), increasing to 20.9 ng/mL within one hour. Laboratory evaluation revealed thrombocytopenia, with a platelet count of 110,000/μL (reference range: 150–400 × 10^3^/μL).

The patient denied recent fever, respiratory symptoms, or substance abuse, and reported only occasional alcohol use. Due to the concerning clinical presentation, she was urgently taken to the cardiac catheterization laboratory. Coronary angiography revealed a patent LAD stent and non‐obstructive coronary artery disease. However, left ventriculography (Video 1) demonstrated features concerning for TCM.

**VIDEO 1:** Left ventriculogram demonstrating apical ballooning consistent with Takotsubo cardiomyopathy. Video content can be viewed at https://onlinelibrary.wiley.com/doi/10.1002/ccr3.71047.

**FIGURE 2:**
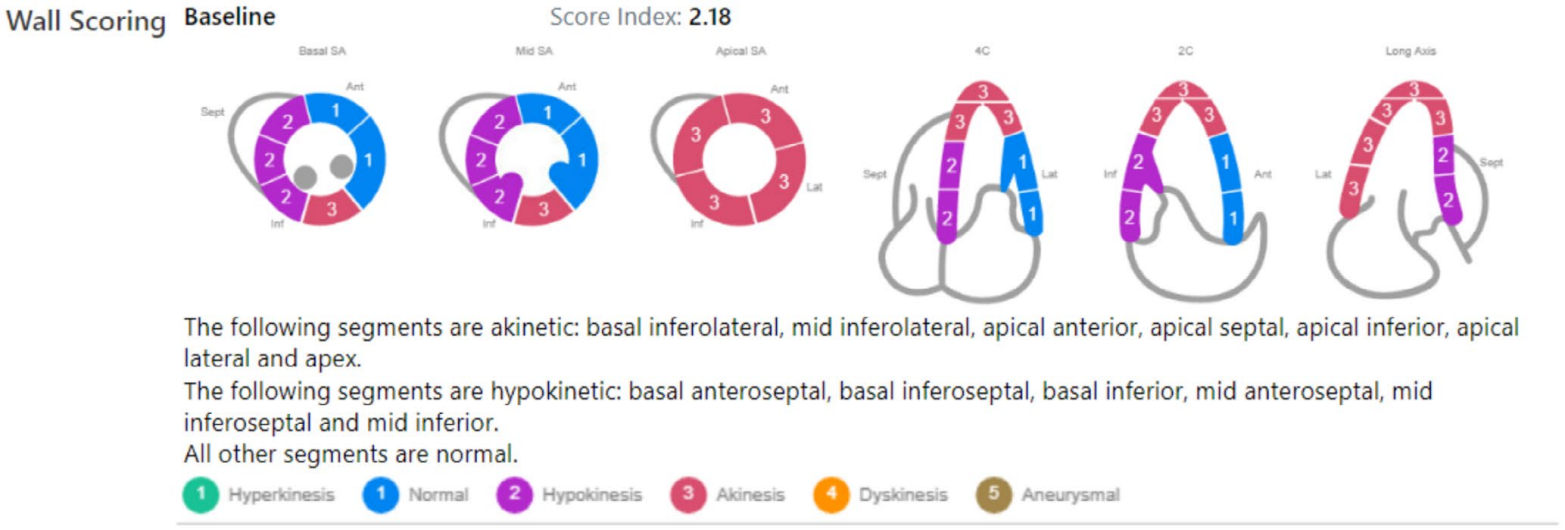
Echocardiographic wall motion scoring demonstrates apical hypokinesis, supporting a diagnosis of Takotsubo cardiomyopathy.

On hospital day two, transthoracic echocardiography revealed a left ventricular ejection fraction (LVEF) of 30%–35%, with regional wall motion abnormalities involving the inferolateral, lateral, and apical walls (Figure 2; Videos 2 and 3).

**VIDEO 2:** Transthoracic echocardiography without contrast demonstrating akinesis of the inferolateral, lateral, and apical walls, consistent with Takotsubo cardiomyopathy. Video content can be viewed at https://onlinelibrary.wiley.com/doi/10.1002/ccr3.71047.

**VIDEO 3:** Transthoracic echocardiography with contrast demonstrating akinesis of the inferolateral, lateral, and apical walls, consistent with Takotsubo cardiomyopathy. Video content can be viewed at https://onlinelibrary.wiley.com/doi/10.1002/ccr3.71047.

**FIGURE 3:**
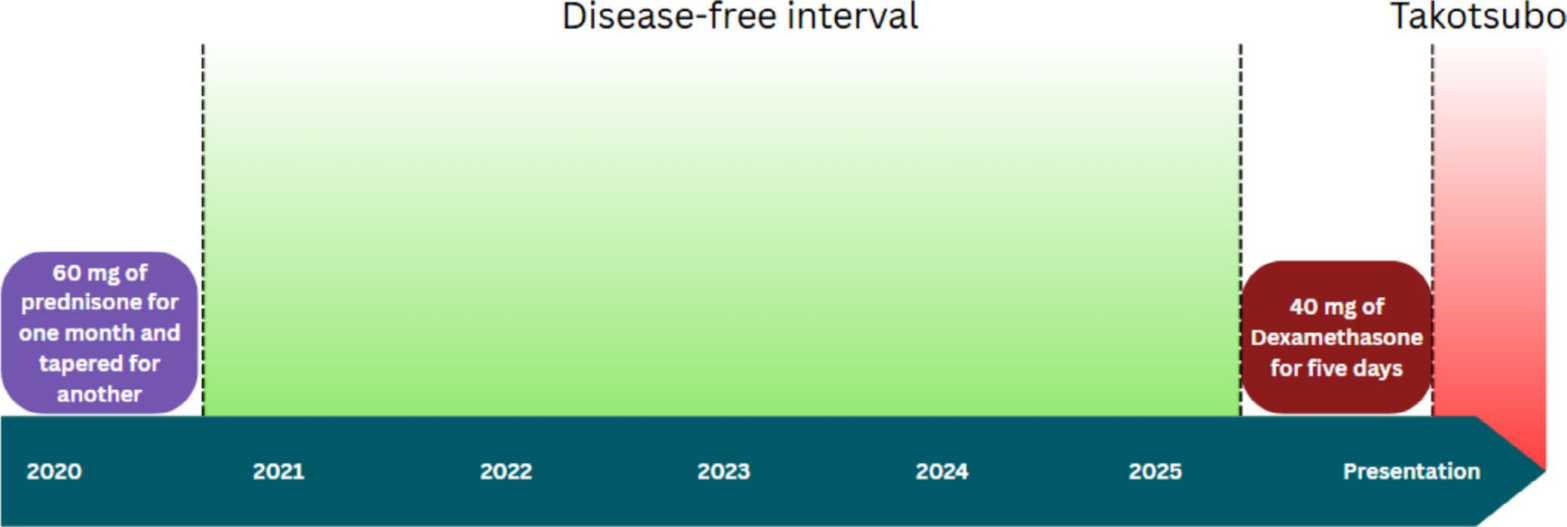
Clinical timeline depicting corticosteroid administration and subsequent onset of Takotsubo cardiomyopathy symptoms.

The patient denied experiencing any recent emotional or physical stressors. However, she had recently completed a five‐day course of high‐dose dexamethasone (40 mg daily) for treatment of ITP, during which her platelet count improved from 17,000/μL to 110,000/μL. Notably, she had previously tolerated prednisone 60 mg daily in 2020, tapered over two months (Figure 3), without similar cardiac symptoms. Hematology advised against further steroid use, and rituximab was initiated for long‐term management of ITP.

During her hospitalization, she experienced brief episodes of non‐sustained ventricular tachycardia. She was initiated on guideline‐directed medical therapy for heart failure with reduced ejection fraction, including sacubitril/valsartan, spironolactone, empagliflozin, and metoprolol succinate. A wearable cardioverter‐defibrillator (LifeVest) was provided. The patient was discharged in stable condition, with close outpatient cardiology follow‐up arranged and a repeat echocardiogram planned in one month to assess recovery of ventricular function.

## 4 Conclusion Results (Outcome and Follow‐Up)

This case reinforces the need to consider high‐dose corticosteroids, such as dexamethasone, as potential non‐traditional triggers of Takotsubo cardiomyopathy. In the absence of overt emotional or physical stressors, clinicians should maintain a high index of suspicion for drug‐induced TCM, particularly in patients with underlying cardiovascular risk. Awareness of this association may prompt early recognition, risk mitigation, and appropriate therapeutic adjustments.

## 5 Discussion

Takotsubo cardiomyopathy (TCM), or “broken heart syndrome,” is a transient left ventricular systolic dysfunction characterized by apical ballooning and the absence of obstructive coronary artery disease. It accounts for 1%–2% of patients presenting with symptoms suggestive of acute coronary syndrome (ACS) [3]. The condition predominantly affects postmenopausal women and often mimics myocardial infarction, with presenting symptoms including chest pain, dyspnea, and elevated cardiac biomarkers. While TCM is classically triggered by emotional or physical stress, a subset of patients develop the condition without any identifiable precipitant.

The pathophysiology of TCM remains incompletely understood, though catecholamine‐mediated myocardial stunning is a leading theory. Elevated serum catecholamine levels, two to three times higher than in patients with ACS, have been observed in TCM, implicating sympathetic overactivation as a key factor [4]. This theory is further supported by reports of TCM in high catecholamine states, such as pheochromocytoma or iatrogenic adrenaline administration [2, 5]. The myocardial apex, which has a higher density of β2‐adrenergic receptors, may be particularly susceptible to catecholamine toxicity [6, 7]. Additional hypotheses include estrogen deficiency–related endothelial dysfunction, which may explain the condition's predominance in postmenopausal women [8, 9].

Our patient developed TCM shortly after receiving high‐dose dexamethasone for immune thrombocytopenic purpura. In the absence of emotional or physical stressors, the timing suggests a pharmacologic trigger. Dexamethasone has been shown to upregulate ornithine decarboxylase activity, leading to increased production of epinephrine and norepinephrine within the adrenal medulla in a dose‐dependent manner [10]. The patient had previously tolerated lower doses of corticosteroids without cardiovascular complications, supporting the hypothesis of a threshold‐dependent catecholaminergic effect contributing to myocardial dysfunction. The prognosis for TCM is generally favorable, with full resolution of apical wall motion abnormalities in the majority of patients, although mortality has been reported in up to 8% of cases [11].

The diagnosis was supported by classic echocardiographic findings, elevated troponin levels, absence of obstructive coronary disease, and exclusion of other stressors or acute illnesses. Her condition improved with guideline‐directed medical therapy for heart failure, and she was discharged with a wearable defibrillator and follow‐up planned for reassessment of cardiac function. This case contributes to the growing body of evidence implicating corticosteroids as potential non‐traditional triggers for TCM, especially in vulnerable cardiovascular populations.

Practical prevention for steroid‐dependent patients. In individuals who require corticosteroids, we recommend (i) using the lowest effective dose and shortest feasible course; (ii) considering steroid‐sparing strategies when appropriate (e.g., TPO‐receptor agonists, rituximab, shared decision‐making on second‐line therapy for ITP); (iii) screening for cardiovascular vulnerability (postmenopausal status, known CAD, prior TCM), avoiding concurrent sympathomimetics when possible, and counseling on symptom vigilance; and (iv) early ECG and troponin testing if chest pain or dyspnea occurs during or within days after high‐dose steroids. No therapy has conclusively prevented TCM recurrence; observational data and consensus statements support guideline‐directed HF therapy when LV dysfunction is present, while the effect of routine β‐blockers or ACEi/ARBs on recurrence remains uncertain [1, 12, 13, 14].

## Author Contributions

**Qutaiba Qafisheh:** conceptualization, project administration, resources, writing – original draft. **Roaa Aljunaidi:** conceptualization, resources, writing – original draft. **Abdalhakim Shubietah:** conceptualization, resources, writing – original draft. **Mohammed AbuBaha:** resources, writing – review and editing. **Hossam Salameh:** data curation, visualization, writing – review and editing. **Muath Baniowda:** data curation, resources. **Mohanad Qwaider:** writing – original draft. **Mohammad Alqadi:** resources. **Imad Hariri:** visualization.

## Ethics Statement

No ethical approval is needed as this is a case report requiring only the consent of the human subjects involved in the study. Ethical Board review is not applicable.

## Consent

Written informed consent was obtained from the patient for publication of this case report and any accompanying images.

## Conflicts of Interest

The authors declare no conflicts of interest.

## Data Availability

Data sharing does not apply to this article as no datasets were generated or analyzed during the current study.
